# Computational design of custom therapeutic cells to correct failing human cardiomyocytes

**DOI:** 10.3389/fsysb.2023.1102467

**Published:** 2023-01-18

**Authors:** Andrew Tieu, Katherine G. Phillips, Kevin D. Costa, Joshua Mayourian

**Affiliations:** ^1^ Cardiovascular Research Institute, Icahn School of Medicine at Mount Sinai, New York, NY, United States; ^2^ Department of Cardiothoracic Surgery, NYU Langone Health, New York, NY, United States; ^3^ Department of Pediatrics, Boston Children’s Hospital, Boston, MA, United States; ^4^ Department of Pediatrics, Harvard Medical School, Boston, MA, United States; ^5^ Department of Pediatrics, Boston University, Boston, MA, United States; ^6^ Department of Pediatrics, Boston Medical Center, Boston, MA, United States

**Keywords:** cell therapy, heart failure, heterocellular coupling, computational modeling, cardiac electrophysiology, action potential, calcium transient

## Abstract

**Background:** Myocardial delivery of non-excitable cells—namely human mesenchymal stem cells (hMSCs) and c-kit^+^ cardiac interstitial cells (hCICs)—remains a promising approach for treating the failing heart. Recent empirical studies attempt to improve such therapies by genetically engineering cells to express specific ion channels, or by creating hybrid cells with combined channel expression. This study uses a computational modeling approach to test the hypothesis that custom hypothetical cells can be rationally designed to restore a healthy phenotype when coupled to human heart failure (HF) cardiomyocytes.

**Methods:** Candidate custom cells were simulated with a combination of ion channels from non-excitable cells and healthy human cardiomyocytes (hCMs). Using a genetic algorithm-based optimization approach, candidate cells were accepted if a root mean square error (RMSE) of less than 50% relative to healthy hCM was achieved for both action potential and calcium transient waveforms for the cell-treated HF cardiomyocyte, normalized to the untreated HF cardiomyocyte.

**Results:** Custom cells expressing only non-excitable ion channels were inadequate to restore a healthy cardiac phenotype when coupled to either fibrotic or non-fibrotic HF cardiomyocytes. In contrast, custom cells also expressing cardiac ion channels led to acceptable restoration of a healthy cardiomyocyte phenotype when coupled to fibrotic, but not non-fibrotic, HF cardiomyocytes. Incorporating the cardiomyocyte inward rectifier K^+^ channel was critical to accomplishing this phenotypic rescue while also improving single-cell action potential metrics associated with arrhythmias, namely resting membrane potential and action potential duration. The computational approach also provided insight into the rescue mechanisms, whereby heterocellular coupling enhanced cardiomyocyte L-type calcium current and promoted calcium-induced calcium release. Finally, as a therapeutically translatable strategy, we simulated delivery of hMSCs and hCICs genetically engineered to express the cardiomyocyte inward rectifier K^+^ channel, which decreased action potential and calcium transient RMSEs by at least 24% relative to control hMSCs and hCICs, with more favorable single-cell arrhythmia metrics.

**Conclusion:** Computational modeling facilitates exploration of customizable engineered cell therapies. Optimized cells expressing cardiac ion channels restored healthy action potential and calcium handling phenotypes in fibrotic HF cardiomyocytes and improved single-cell arrhythmia metrics, warranting further experimental validation studies of the proposed custom therapeutic cells.

## 1 Introduction

Cardiovascular disease, including heart failure (HF), remains a leading cause of morbidity and mortality worldwide (Virani et al., 2021). In an aging and growing population, the prevalence of HF continues to increase (Virani et al., 2021). Substantial advancements have been made to reduce mortality and treat HF with pharmacologic (e.g., angiotensin-converting enzyme inhibitors, β-blockers) and device (e.g., coronary revascularization, implantable cardioverter-defibrillators, and cardiac resynchronization) interventions (Virani et al., 2021). Further pharmacological advancements (e.g., Entresto and SGLT-2 inhibitors) also help prevent adverse remodeling; but to date, no available therapy can restore healthy function to failing myocardium, motivating alternative treatments such as cell-based therapies.

Cell-based therapies, including delivery of human mesenchymal stem cells (hMSCs) and c-kit^+^ cardiac interstitial cells (hCICs), are considered safe and purported to protect or even repair diseased myocardium (Sanganalmath and Bolli, 2013; Braunwald, 2022). Cardiac cell therapies represent promising approaches to treat HF that are being actively investigated in clinical trials. For example, in the phase II MSC-HF trial, patients with ischemic HF had improved left ventricular function at 12-month follow-up (Mathiasen et al., 2015). Subsequently, the CONCERT-HF phase II trial showed hCICs alone and hCICs combined with hMSCs reduced HF-related major adverse events (Bolli et al., 2021). These modestly beneficial findings motivate efforts to maximize therapeutic efficacy by enhancing and refining cell-based treatment strategies for myocardial diseases through rational design.

Recent work has boldly attempted to create “enhanced” engineered cells derived from hCICs and/or hMSCs (Mohsin et al., 2012; Quijada et al., 2015; Broughton et al., 2019; Firouzi et al., 2020). For example, Quijada et al. showed that fused hCIC-hMSC CardioChimeras were superior to hCICs alone, hMSCs alone, or their combination in a single suspension (Quijada et al., 2015). Others have demonstrated the promise of delivering non-excitable cells genetically engineered to express specific ion channels; for example, Nguyen et al. demonstrated that primary human fibroblasts engineered to express small prokaryotic sodium channels could rescue slowed conduction in an *in vitro* model of cardiac interstitial fibrosis (Nguyen et al., 2016), and Yankelson et al. showed that injecting genetically engineered fibroblasts expressing potassium channels into the rat right ventricle prevented induction of ventricular arrhythmias *in vivo* (Yankelson et al., 2008).

Our lab has recently created a computational pipeline to investigate the heterocellular coupling effects of hMSCs (Mayourian et al., 2016; Mayourian et al., 2017; Mayourian et al., 2018a; Mayourian et al., 2018b), hCICs (Phillips et al., 2021), and “enhanced” CardioChimeras (Phillips et al., 2021) on healthy and failing cardiomyocytes. In our series of works, we: 1) developed hMSC (Mayourian et al., 2016) and hCIC (Phillips et al., 2021) models that were validated by previously published experimental data (Li et al., 2005; Zhang et al., 2014); 2) helped unravel relative paracrine *versus* heterocellular coupling effects of delivered therapeutic cells on human cardiac contractility and arrhythmogenicity (Mayourian et al., 2017); and 3) demonstrated that heterocellular coupling of standard hMSCs and hCICs are insufficient to correct failing human cardiomyocytes (Phillips et al., 2021).

In this study, we leverage our easily adjustable computational modeling tool—capable of theoretically screening a large set of candidate genetically engineered cells expressing specific ion channels—to explore the therapeutic potential of delivering “enhanced” customizable engineered cells to failing cardiomyocytes. More specifically, we test the hypothesis that custom hypothetical cells can be designed to restore a healthy phenotype when heterocellularly coupled to human HF cardiomyocytes. Using a genetic model fitting algorithm, we investigate whether customized hypothetical cells composed of non-excitable ion channels, as well as cardiomyocyte channels, can correct the failing action potential (AP) and calcium transient (CaT) waveforms back to healthy conditions. We identify key ion channels required to generate custom cells that satisfy the corrective acceptance criteria, and simulate the translational potential of genetically engineering hMSCs and hCICs to promote restoration of failing cardiomyocytes. Finally, we explore the potential adverse effects (e.g., arrhythmogenicity) of our accepted custom cells on failing cardiomyocytes to more fully examine the translational potential of such an approach.

## 2 Materials and methods

All data, code, methods, and study materials are available upon request by contacting the corresponding authors.

### 2.1 Healthy and failing cardiac electrophysiology models

Healthy human cardiomyocytes (hCMs) were modeled using the O’Hara et al. endocardial ventricular model (O'Hara et al., 2011) with sodium current dynamics modified as detailed by Mora et al. (Mora et al., 2018). To model non-fibrotic HF cardiomyocytes, several parameters were modified as detailed elsewhere by Mora et al. (Mora et al., 2018) to reflect the underlying pathophysiology of HF with reduced ejection fraction. HF in fibrotic myocardium was modeled as coupling of an individual HF with reduced ejection fraction cardiomyocyte to 5 fibroblasts with a gap junctional conductance of 1 nS, as detailed in our previous work (Phillips et al., 2021) (adapted from Mora et al. (Mora et al., 2018)). Pacing frequency was set at 1 Hz, and all simulations were run for 500 beats to achieve a steady state solution, in accordance with our previous work (Phillips et al., 2021).

### 2.2 Generating custom cell models expressing non-excitable ion channels

Theoretical candidate non-excitable custom cells were developed for the correction of failing cardiomyocytes *via* heterocellular coupling, expressing membrane channels from hCICs, hMSCs, and cardiac fibroblasts (CFs).

Ionic currents from hCIC channels—including a sodium current, inward rectifying potassium current, large-conductance calcium-activated potassium current, and transient outward current—were modeled as described in our recent work (Phillips et al., 2021). Ionic currents from hMSC channels—including a transient outward current, sodium current, L-type calcium current, calcium-activated potassium current, and delayed rectifier potassium current—were modeled as described in our previous work (Mayourian et al., 2016). Finally, ion currents from CF channels—including the delayed rectifier potassium current, inward rectifier potassium current, and sodium/potassium pump current—were modeled as described elsewhere (MacCannell et al., 2007). Total ionic flow through the custom cell was subsequently calculated as the sum of the ionic flows through hCIC, hMSC, and CF channels, where each channel’s maximal conductance was scaled as described below as per our genetic algorithm.

The capacitance of all custom cells was set to 40pF, to reflect the approximate capacitance of hMSCs (Mayourian et al., 2016) and hCICs (Phillips et al., 2021), and to reflect their smaller size compared to hCMs (O'Hara et al., 2011). During simulations, intracellular ion concentrations of all custom cells were assumed to be held constant.

### 2.3 Generating custom cell models expressing cardiomyocyte ion channels

Another type of theoretical candidate custom cell was developed that expressed healthy hCM membrane channels in addition to the previously described non-excitable ion channels.

Ionic currents from healthy hCM channels—including the fast sodium current, late sodium current, transient outward current, L-type calcium current, rapid delayed rectifier potassium current, slow delayed rectifier potassium current, and inward rectifier potassium current—were modeled as described in Mora’s modification (Mora et al., 2018) of the O’Hara et al. human endocardial ventricular cardiomyocyte model (O'Hara et al., 2011). Total ionic flow through the custom cell was subsequently calculated as the sum of ionic flows through hCIC, hMSC, CF, and hCM channels, where each channel’s maximal conductance was scaled as described below as per our genetic algorithm.

Similar to above, the capacitance of all custom cells was set to 40pF, and the intracellular ion concentrations were held constant.

### 2.4 Simulating heterocellular coupling

A human cardiomyocyte (non-fibrotic HF, fibrotic HF, or healthy) was coupled to custom cells using the following pair of equations, similar to our previous work (Phillips et al., 2021) and as described elsewhere (Xie et al., 2009):

$$-C_{CM}\frac{dV_{CM}}{dt}=I_{ion,\;CM}+n_{custom}G_{gap}\left(V_{CM}-V_{custom}\right)$$ (1)

$$-C_{custom}\frac{dV_{custom}}{dt}=I_{ion,\;custom}+G_{gap}\left(V_{custom}-V_{CM}\right)$$ (2)

where: C_CM_ and C_custom_ represent the membrane capacitance of the cardiomyocyte and custom cell, respectively; V_CM_ and V_custom_ represent the membrane voltage of the cardiomyocyte and custom cell, respectively; t is time; I_ion,CM_ and I_ion,custom_ represent total ionic flow through the cardiomyocyte and custom cell, respectively; n_custom_ is the integer number of coupled custom cells per cardiomyocyte; and G_gap_ is gap junctional conductance between the cells. Custom cells were only coupled to cardiomyocytes (i.e., custom cells were not also coupled to fibroblasts).

### 2.5 Genetic algorithm

A genetic algorithm for parallel computational model fitting was implemented as described elsewhere (Groenendaal et al., 2015; Devenyi et al., 2017). An initial population of 2500 custom cell models was generated for each *in silico* experiment with varying ion channel maximal conductances. Maximal conductance parameters for each ion channel were defined as the original value multiplied by a scaling factor obtained by pseudo-random sampling from a uniform distribution ranging from 0.01% to 1000%. Gap junction conductance between the custom cell and the failing cardiomyocyte was similarly obtained by pseudo-random sampling from a uniform distribution ranging from 0.01% to 1000% of its designated baseline value of 1 nS. The upper limit for gap junction conductance was selected to minimize computational cost, as our recent work showed that when coupling cardiomyocytes to non-excitable cells, gap junctional conductance values greater than 10 nS had minimal additional effect on AP and CaT waveforms (Phillips et al., 2021). The scaled number of heterocellular coupled custom cells per cardiomyocyte was pseudo-randomly sampled from a discrete uniform distribution ranging from 1 to 5 (baseline value of one heterocellular coupled custom cell per cardiomyocyte). The upper limit of 5 was selected as a practical consideration of therapeutic delivery. For each custom cell model, the AP and CaT waveforms were extracted from the final (i.e., 500th) simulated beat.

Subsequent to model creation, the genetic algorithm was run for 5 rounds, or “generations”, aiming to achieve at least 10% acceptance of custom cells. Further rounds were purposely not pursued, as the solution space would further converge (whereas a wide solution space was of interest in this study). Each generation of the genetic algorithm consisted of 4 steps: selection, crossover, mutation, and elitism (Bot et al., 2012). During selection, models were randomly paired, and the AP and CaT root mean square errors (RMSE_AP_ and RMSE_CaT_, respectively) between the custom cell-treated HF cardiomyocyte and a healthy cardiomyocyte for the first 500 ms of the final beat were defined as the objective functions. All RMSEs were normalized to the RMSE between a healthy and untreated failing cardiomyocyte. The model achieving a greater number of objective function values less than 50% (where RMSE_AP_ is the first objective function and RMSE_CaT_ is the second objective function) was kept. In the case of a tie, the model with the lower overall percent RMSE between the two waveforms was chosen. Selection was completed twice per generation such that the resulting number of models advancing remained at 2500, with each original model advancing either 0, 1, or 2 times post-selection. After selection, models that advanced were randomly paired once more for crossover. Each pair had a 90% chance of undergoing crossover, in which each parameter value then had a 50% chance of crossing over or exchanging between models. After crossover, each model parameter had a 1% chance of undergoing random mutation, where it would be scaled by a log-normal distribution with a standard deviation of 20% such that >99.9% of mutations would fall between a 0.5-fold and 2-fold change. Finally, an elitism strategy was employed such that the top 10% of models from each generation replaced the bottom 10%.

### 2.6 Custom cell acceptance criteria

Following genetic algorithm adjustment, accepted models from the final generation were determined based on AP and CaT as compared to healthy cardiomyocytes. Specifically, models in the final generation were accepted if RMSE_AP_ and RMSE_CaT_ in the first 500 ms of the final beat were both less than 50% of their respective RMSEs between a healthy and untreated HF cardiomyocyte.

### 2.7 Post hoc analyses

Following genetic algorithm adjustment, several metrics for electrophysiology and calcium handling in accepted models were calculated, including action potential duration at 50% (APD_50_) and 90% (APD_90_) repolarization, peak membrane voltage, maximum upstroke velocity, resting membrane potential, maximum systolic calcium concentration, maximum diastolic calcium concentration, calcium transient amplitude, and calcium relaxation time constant at 50% (τ_50_) and 90% (τ_90_) decay. These metrics were used to further interrogate the effects of custom cell coupling. Note statistical analyses were deferred given the nature of this work whereby arbitrarily large sample sizes could be achieved, which is in accordance with our previous studies (Mayourian et al., 2016).

## 3 Results

### 3.1 Custom cells expressing non-excitable channels are unable to correct the human heart failure cardiomyocyte phenotype

First, to investigate whether custom cells with non-excitable cell channels can restore a healthy hCM phenotype, we created a population of 2500 custom cell models that variably express ion channels from hCICs, hMSCs, and CFs. The genetic algorithm was subsequently used to promote selection of models that restore failing electrical and calcium cycling behavior back towards a healthy phenotype.

**FIGURE 1:**
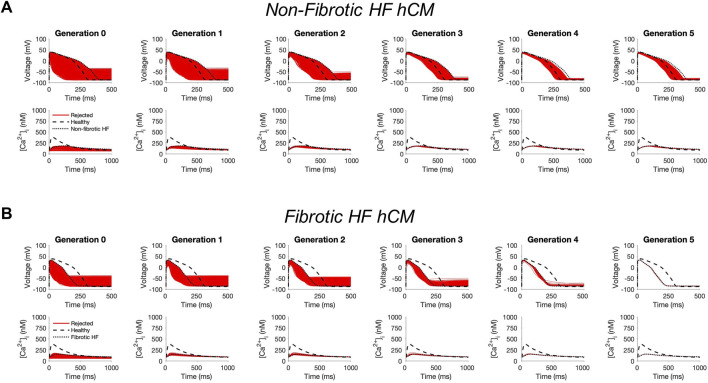
Genetic algorithm-based approach to identify custom cells expressing non-excitable cell channels are unable to restore failing cardiomyocyte phenotype. Initial populations of 2500 custom cell models were generated for heterocellular coupling with non-fibrotic **(A)** and fibrotic **(B)** human heart failure (HF) cardiomyocytes with pseudo-random perturbations of non-excitable cell ion channel maximal conductivities, gap junction conductances, and number of coupled cells. Custom cell populations underwent 5 generations of genetic algorithm evolution. Custom cells were accepted if root mean square error was less than 50% relative to untreated HF cardiomyocytes for both action potential and calcium transient waveforms; in this set of simulations, all models were outside this range and were therefore rejected (red). Waveforms for healthy human cardiomyocytes (hCMs) and untreated HF cardiomyocytes are indicated by dashed and dotted lines, respectively.

In non-fibrotic HF cardiomyocytes, heterocellular coupling with the population of custom cells led to no acceptable solutions (Figure 1A). Similarly, restoration was unable to be achieved in fibrotic HF cardiomyocytes (Figure 1B). While custom cell coupling was able to correct AP waveforms in a majority (90%, or 2245/2500) of non-fibrotic HF models in the genetic algorithm final generation, it was unable to correct AP waveforms (0%, or 0/2500) in the fibrotic HF models. CaT was not restored in either HF condition.

### 3.2 Custom cells expressing excitable cell channels correct the human heart failure cardiomyocyte phenotype

**FIGURE 2:**
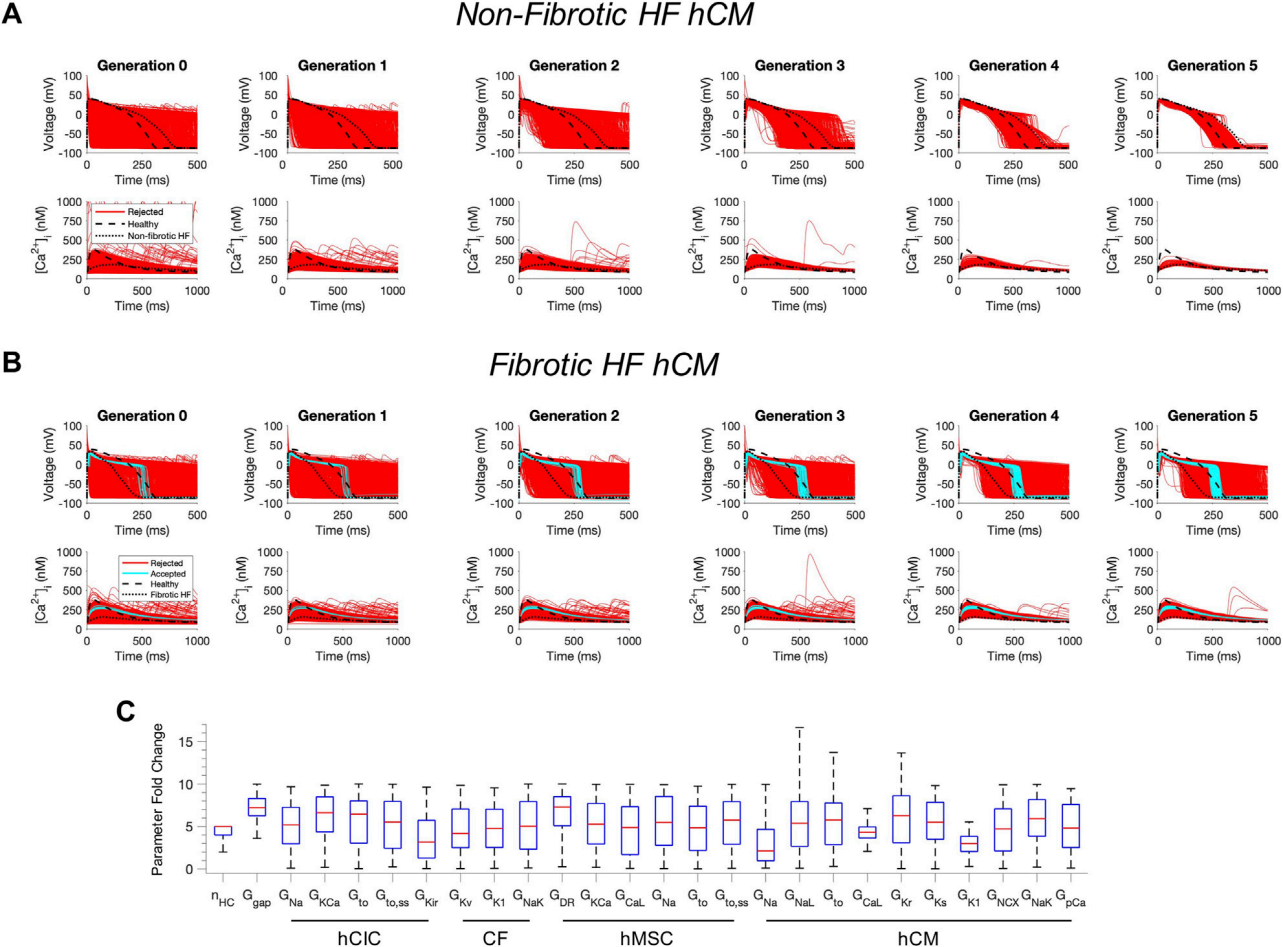
Custom cells expressing excitable cell channels restore a healthy phenotype in fibrotic failing cardiomyocytes. Initial populations of 2500 custom cell models were generated for heterocellular coupling with non-fibrotic **(A)** and fibrotic **(B)** heart failure (HF) human cardiomyocytes (hCMs) with pseudo-random perturbations of gap junction conductances, number of coupled cells, and both cardiomyocyte and non-excitable cell ion channel maximal conductivities. Custom cell populations underwent 5 generations of genetic algorithm evolution, whereby custom cells were accepted if root mean square error was less than 50% for both action potential and calcium transient waveforms (blue); models outside this range were rejected (red). Waveforms for healthy and untreated HF cardiomyocytes are indicated by dashed and dotted lines, respectively. **(C)** Distribution of accepted custom cell model parameter fold changes (relative to baseline values) in the fibrotic HF condition shown as box and whisker plots. Note for certain parameters, a fold-change >10 is achieved due to the genetic algorithm mutation step. Abbreviations: hMSCs, human mesenchymal stem cells; hCIC, c-kit^+^ cardiac interstitial cells; CF, cardiac fibroblast; hCM, human cardiomyocyte; n_HC_, Number of heterocellularly coupled cells; G_gap_, Gap junction conductance; G_Na_, Na^+^ channel conductance; G_KCa_, Large-conductance Ca^2+^-activated K^+^ channel conductance; G_to_, Transient outward K^+^ channel conductance; G_to,ss_, Transient outward K^+^ channel steady-state conductance; G_Kir_, Inward rectifying K^+^ channel conductance; G_Kv_, Time- and voltage-dependent K^+^ channel conductance; G_K1_, Inward rectifier K^+^ channel conductance; G_NaK_, Na^+^/K^+^ pump activity; G_DR_, Delayed rectifier K^+^ channel conductance; G_CaL_, L-type Ca^2+^ channel conductance; G_NaL_, Na^+^ channel conductance, late component; G_Kr_, Rapid delayed rectifier K^+^ channel conductance; G_Ks_, Slow delayed rectifier K^+^ channel conductance; G_NCX_, Na^+^/Ca^2+^ exchanger conductance; G_pCa_ Sarcolemmal Ca^2+^ pump activity.

Next, we tested whether custom cells expressing channels from cardiomyocytes can restore a healthy phenotype. To do so, we created a population of 2500 custom cell models that variably express ion channels from healthy hCMs, in addition to hCICs, hMSCs, and CFs (Figure 2). The genetic algorithm was subsequently used to promote selection of models that restore electrical and calcium cycling behavior back towards a healthy phenotype.

The addition of excitable cell channels to custom cells was insufficient to restore both a healthy AP and CaT cardiac phenotype in non-fibrotic HF cardiomyocytes (Figure 2A). While 1963/2500 (79%) custom cells met acceptance criteria for the AP, only 4/2500 (0.2%) of the custom cells had <50% RMSE_CaT_ (Figure 2A), whereby the CaT peak was restored by as much as 55% from non-fibrotic HF to healthy conditions.

**FIGURE 3:**
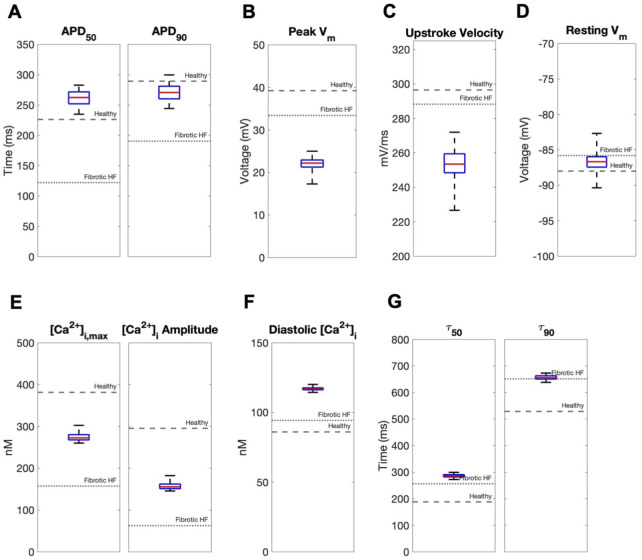
Effects of accepted custom cell heterocellular coupling on fibrotic heart failure cardiomyocyte electrophysiology and calcium handling. Action potential and calcium transient metrics of fibrotic heart failure (HF) cardiomyocytes when coupled to accepted custom cells are shown as box and whisker plots. Action potential metrics include: **(A)** Action potential duration (APD) at 50% repolarization (APD_50_) and 90% repolarization (APD_90_); **(B)** peak voltage; **(C)** upstroke velocity; and **(D)** resting membrane potential. Calcium transient metrics include: **(E)** peak intracellular calcium and calcium transient amplitude; **(F)** diastolic calcium; and **(G)** calcium relaxation time constant at 50% decay (τ_50_) and 90% decay (τ_90_). Values for untreated healthy and fibrotic HF cardiomyocytes are shown as dashed and dotted lines, respectively.

In contrast, coupling a population of custom cells to fibrotic HF cardiomyocytes led to solutions that satisfied acceptance criteria for both AP and CaT within Generation 0 (Figure 2B). By Generation 5, 266/2500 (11%) custom cells met the acceptance criteria (Figure 2B). Notably, the AP morphology of accepted solutions differed from healthy waveforms, whereby the upstroke velocity and peak voltage appeared lower, phase 2 was flatter, and phase 3 repolarization was more rapid (Figure 2B). In addition, the accepted CaT appeared to have lower calcium amplitude, higher diastolic calcium, and slower relaxation than healthy hCMs (Figure 2B). Figure 3 corroborates these findings and quantifies the differences in standard AP and CaT waveform metrics for treated HF fibrotic cardiomyocytes in comparison to healthy and untreated fibrotic HF hCMs.

Distributions of accepted custom cell parameters are shown in Figure 2C. Accepted solutions were dependent on gap junction conductance and number of coupled custom cells parameters, which spanned a narrow range with median values of 7.2 [IQR 6.3-8.3] and 5 [IQR 4-5], respectively. While a majority of ion channel maximal conductance parameters spanned the entire search space, hCM L-type calcium current (I_CaL_) and inward rectifier K^+^ current (I_K1_) spanned a narrow range in order to correct the fibrotic HF phenotype with a median maximal conductance scaling of 4.3 [IQR 3.6-5.0] and 3.0 [IQR 2.1-3.8], respectively (Figure 2C).

### 3.3 Exploring key properties of accepted custom cells

Next, we sought to understand the key properties of accepted custom cells that led to restoration of failing fibrotic HF cardiomyocytes in Figure 2. Based on the narrow solution space for select parameters in Figure 2C, we hypothesized I_CaL_ and I_K1_ were critical for phenotype restoration of failing cardiomyocytes. To test this, we repeated the genetic algorithm from Figure 2, but without I_CaL_ and I_K1_ as candidate channels for the custom cell (Supplementary Figure S1). Indeed, in this case, no custom cells met the acceptance criteria (Supplementary Figure S1), suggesting either or both of these select channels (i.e., I_CaL_ and/or I_K1_) are critical to creating custom cells that can effectively correct the fibrotic HF phenotype. We next created a population of 2500 custom cells including only the following parameters of interest: I_CaL_, I_K1_, gap junction conductance, and number of coupled custom cells.

**FIGURE 4:**
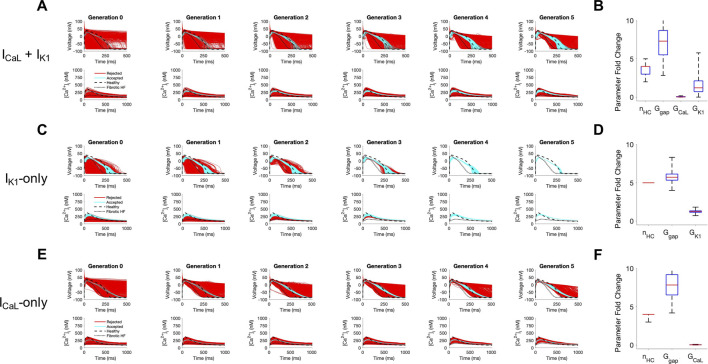
Custom cells expressing cardiomyocyte L-type calcium and inward rectifier channels are capable of correcting fibrotic failing cardiomyocytes. Initial populations of 2500 custom cell models were generated expressing **(A)** L-type calcium (I_CaL_) and inward rectifier (I_K1_) channels; **(C)** I_K1_ only; and **(E)** I_CaL_ only with pseudo-random perturbations to gap junction conductances, number of heterocellularly coupled custom cells to fibrotic heart failure (HF) cardiomyocytes, and custom cell ion channel maximal conductances. Custom cell populations underwent 5 generations of genetic algorithm evolution, whereby custom cells were accepted if root mean square error was less than 50% for both action potential and calcium transient waveforms (blue); models outside this range were rejected (red). Distributions of accepted custom cell model parameter fold changes (relative to baseline values) in the fibrotic HF condition are shown as box and whisker plots for **(B)** I_CaL_ and I_K1_, **(D)** I_K1_, and **(F)** I_CaL_. Note for certain parameters, a fold-change >10 is achieved due to the genetic algorithm mutation step. Waveforms for healthy and untreated HF cardiomyocytes are indicated by dashed and dotted lines, respectively. Abbreviations: n_HC_, Number of heterocellularly coupled cells; G_gap_, Gap junction conductance; G_CaL_, L-type Ca^2+^ channel conductance; G_K1_, Inward rectifier K^+^ channel conductance.

As shown in Figure 4, coupling custom cells that incorporate only these 4 parameters to fibrotic HF cardiomyocytes satisfied acceptance criteria for both AP and CaT in Generation 0 (Figure 4A). By Generation 5, 579/2500 (23%) custom cells met acceptance criteria (Figure 4A). The AP morphology of accepted solutions more closely resembled healthy waveforms in comparison to custom cells from Figure 2B where all channels were candidates; this is corroborated by the RMSE, where Figure 2B custom cells with all channels as candidates had a median RMSE_AP_ of 0.45 [IQR 0.43-0.47] and median RMSE_CaT_ of 0.47 [IQR 0.45-0.49], in comparison to Figure 4A custom cells with select channels (i.e., I_K1_ and I_CaL_) which had a median RMSE_AP_ of 0.37 [IQR 0.32-0.41] and median RMSE_CaT_ of 0.43 [IQR 0.40-0.45] (Supplementary Figure S2). While the acceptance bounds for I_K1_, gap junction conductance, and number of coupled custom cells were nearly unchanged when considering select channels (Figure 4B) rather than all channels (Figure 2C), interestingly, I_CaL_ had a lower and more restricted acceptance range (Figure 4B); this could be explained by I_CaL_ not having to counteract a wide range of repolarization currents which were previously included as candidate channels.

We next explored if incorporating only I_CaL_, or only I_K1_, would be sufficient to create accepted custom cells. As shown in Figure 4C, when incorporating only I_K1_ as a candidate channel, accepted custom cells closely resembled incorporating both I_CaL_ and I_K1_ with a similar RMSE (Supplementary Figure S2) and acceptance parameter range (Figure 4D). In comparison, when incorporating only I_CaL_ as a candidate channel (Figure 4E), accepted custom cells had a larger RMSE (Supplementary Figure S2) despite a similar acceptance bound (Figure 4F). The corresponding raw accepted parameter solutions for ion channels and their relevant experimental metrics are shown in Supplementary Table S1. Interestingly, the I_CaL_-only custom cell solution had minimal I_CaL_ activity, and appeared to resemble a passive-cell type; however, running the genetic algorithm on passive cells alone led to no accepted solutions (Supplementary Figure S3). Altogether, these findings suggest cardiomyocyte inward rectifier K^+^ channel is most critical to accomplish the phenotypic rescue.

### 3.4 Custom cells expressing only the cardiomyocyte inward rectifier channel improves single-cell metrics associated with arrhythmogenesis

**FIGURE 5:**
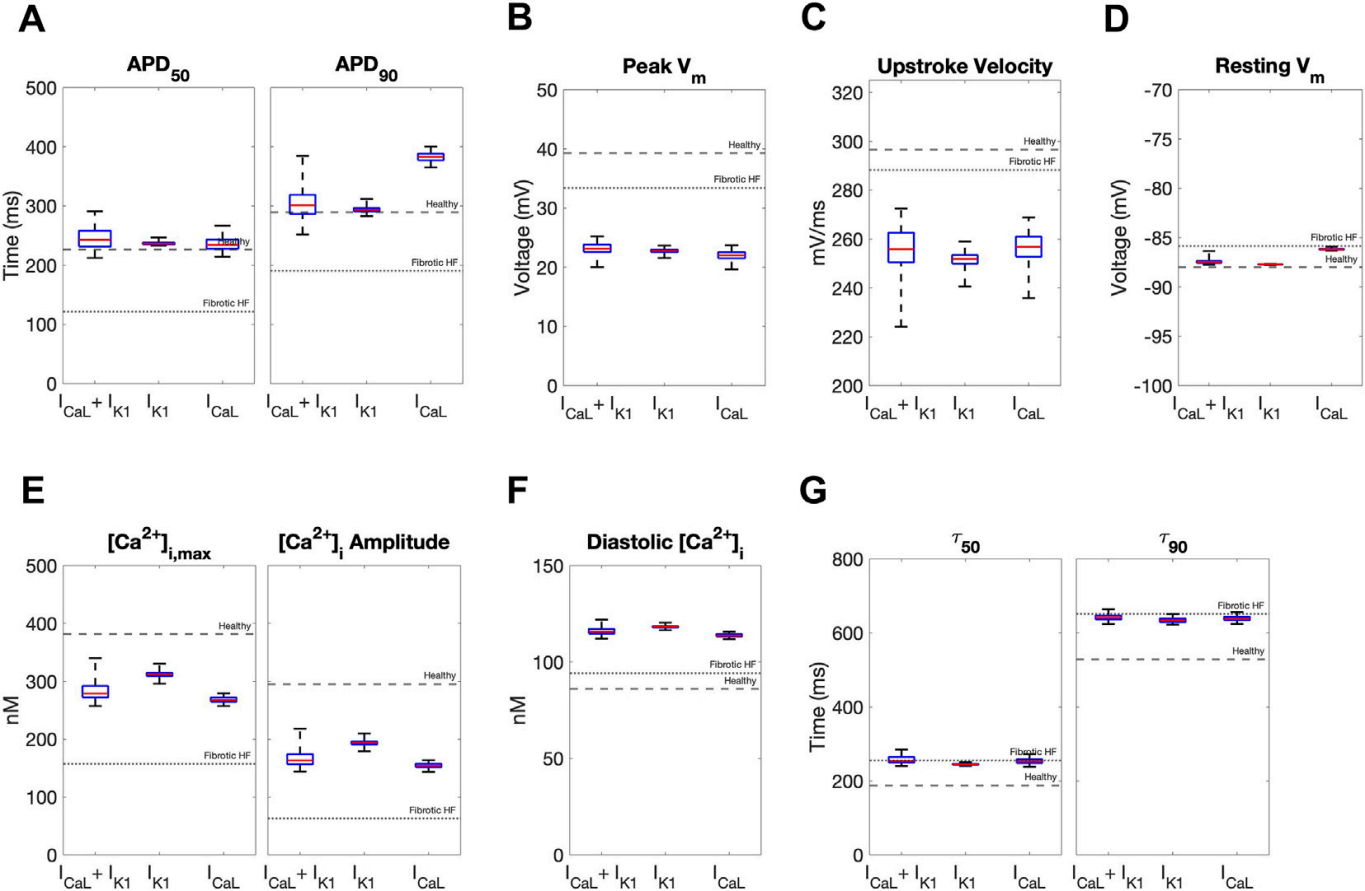
Effects of accepted custom cell heterocellular coupling on fibrotic heart failure cardiomyocyte electrophysiology and calcium handling. Action potential and calcium transient metrics are shown as box and whisker plots for coupling fibrotic heart failure (HF) cardiomyocytes to accepted custom cells with cardiomyocyte L-type calcium channel (I_CaL_), inward rectifier channel (I_K1_), or both (I_CaL_ + I_K1_). Action potential metrics include: **(A)** Action potential duration (APD) at 50% repolarization (APD_50_) and 90% repolarization (APD_90_); **(B)** peak voltage; **(C)** upstroke velocity; and **(D)** resting membrane potential. Calcium transient metrics include: **(E)** peak intracellular calcium and calcium transient amplitude; **(F)** diastolic calcium; and **(G)** calcium relaxation time constant at 50% decay (τ_50_) and 90% decay (τ_90_). Values for healthy and untreated fibrotic HF cardiomyocytes are shown as dashed and dotted lines, respectively.

We next calculated single-cell AP and CaT metrics associated with arrhythmogenesis to explore potential adverse risks in our accepted custom cells incorporating I_CaL_-only, I_K1_-only, or both. The similarities between incorporating I_K1_ only *versus* both I_K1_ and I_CaL_ into custom cells were further confirmed by these metrics (Figure 5). In contrast, as shown in Figure 5, the custom cell only incorporating I_CaL_ had a longer APD_90_ and increased APD triangulation (i.e., APD_90_ minus APD_50_) compared to I_K1_-only and I_K1_+I_CaL_ custom cells, which are surrogate markers of pro-arrhythmia. All custom cells had similarly decreased upstroke velocity, a surrogate marker of conduction velocity. Finally, I_CaL_ had a depolarized resting membrane potential compared to I_K1_-only and I_K1_+I_CaL_ custom cells, also a surrogate marker of pro-arrhythmia. Altogether, these single-cell metrics suggest custom cells incorporating only I_CaL_ may pose a greater risk of an arrhythmogenic substrate compared to I_K1_-only and I_K1_+I_CaL_ custom cells, further supporting the incorporation of cardiomyocyte I_K1_ only in custom cells.

### 3.5 Theoretical mechanism of correcting the failing cardiomyocyte phenotype

**FIGURE 6:**
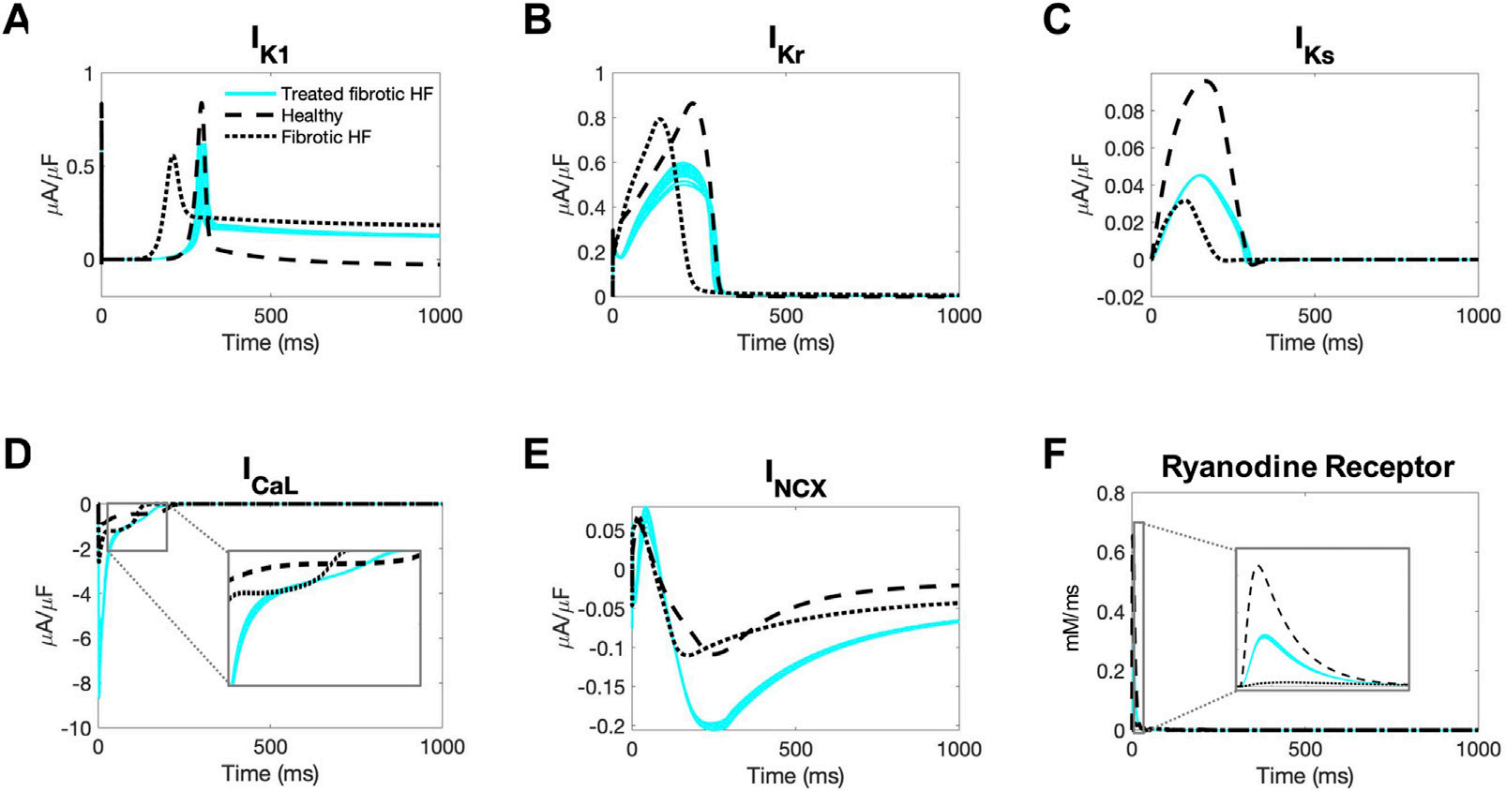
Ionic currents of fibrotic heart failure cardiomyocytes during heterocellular coupling with accepted custom cells. Plots of the following ionic currents of fibrotic heart failure (HF) cardiomyocytes when coupled to 25 randomly selected accepted custom cells (blue) that express cardiomyocyte inward rectifier channel: **(A)** inward rectifier current (I_K1_); **(B)** rapid delayed rectifier (I_Kr_); **(C)** slow delayed rectifier (I_Ks_); **(D)** L-type calcium (I_CaL_); **(E)** sodium-calcium exchanger (I_NCX_); **(F)** ryanodine receptor. Zoomed in views of currents for I_CaL_ and ryanodine receptor are inset. Healthy and untreated fibrotic HF cardiomyocytes are shown as dashed and dotted lines, respectively.

Next, we aimed to gain insight into the mechanisms by which our accepted custom cells were theoretically able to restore healthy AP and CaT waveforms in fibrotic HF cardiomyocytes. To do so, we first explored individual cardiomyocyte ionic currents during the action potential when coupled to 25 randomly selected accepted custom cell solutions (Figure 6).

As shown in Figure 6A, inward rectifier activity of failing cardiomyocytes when coupled to accepted custom cells were more representative of healthy cardiomyocytes; delayed rectifier activity was intermediate to untreated fibrotic HF cardiomyocytes and healthy cardiomyocytes (Figures 6B, C). Interestingly, as shown in Figure 6D, L-type calcium current was more robust in fibrotic HF cells treated with accepted custom cells. Given that this occurred as early as phase 0 of the action potential, this could likely be explained by achieving a favorable resting membrane potential to maximize channel kinetics. In addition, the later activity of the L-type calcium channel is intermediate to fibrotic HF cardiomyocytes and healthy cardiomyocytes, likely in the setting of prolonging phase 2 (Figure 6D inset). By increasing L-type calcium channel activity altogether, this is presumably increasing calcium-induced calcium release (with increased ryanodine receptor activity in phase 0 as shown in Figure 6F). This is further corroborated by increased sodium-calcium exchanger activity during repolarization indicative of more intracellular calcium being extracted (Figure 6E).

To gain further insight, we also explored the membrane voltage and gap junctional current for the same randomly selected accepted custom cells during the cardiomyocyte action potential (Supplementary Figure S4). As shown in Supplementary Figure S4C, the accepted custom cell acts predominantly as an electrical sink during phases 0, 1, late 3, and 4. In doing so, the peak and plateau membrane voltages of the cardiomyocyte are reduced (Supplementary Figure S4A), which may be more favorable for achieving larger magnitude L-type calcium channel currents based on the established current-voltage relationship (O'Hara et al., 2011), which may in turn also contribute to improved calcium handling.

### 3.6 Translational relevance: Creating custom engineered hMSCs and hCICs

Finally, given that custom cells expressing I_K1_ alone showed promise for correcting fibrotic HF cardiomyocyte phenotype with minimized RMSE and AP single-cell markers of arrhythmogenesis, we explored the translational potential of genetically engineering standard cell-based cardiotherapeutics—namely hMSCs and hCICs—to express this cardiomyocyte channel.

**FIGURE 7:**
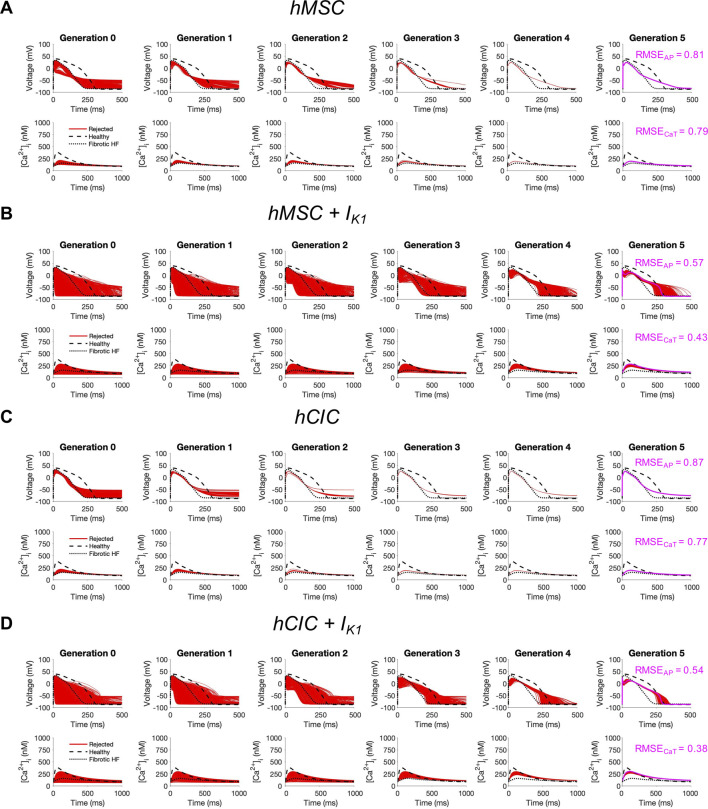
Custom engineered human mesenchymal stem cells and c-kit^+^ cardiac interstitial cells expressing cardiomyocyte inward rectifier channels reduce root mean square error. Initial populations of 2500 **(A)** control human mesenchymal stem cells (hMSCs), **(B)** hMSCs expressing cardiomyocyte inward rectifier channel (I_K1_), **(C)** control c-kit^+^ cardiac interstitial cells (hCICs), and **(D)** hCICs expressing I_K1_ were generated for heterocellular coupling with fibrotic heart failure (HF) cardiomyocytes with pseudo-random perturbations of gap junction conductances, number of coupled cells, and I_K1_ maximal conductance. Each population underwent 5 generations of genetic algorithm evolution, whereby cells were accepted if root mean square error (RMSE) was less than 50% for both action potential (AP) and calcium transient (CaT) waveforms; all models outside this range were rejected (red). The minimum total RMSE_AP_ + RMSE_CaT_ achieved for each group is shown in pink. Waveforms for healthy and untreated HF cardiomyocytes are indicated by dashed and dotted lines, respectively.

To test this, we started with either hMSCs or hCICs, and varied the following parameter variables: number of cells, gap junction conductance, and I_K1_ maximal conductance. As shown in Figure 7, while no candidate cells were accepted, the minimum RMSE_AP_ of hMSCs decreased from 81% to 57% when expressing I_K1_, and the minimum RMSE_CaT_ decreased from 79% to 43% when expressing I_K1_ (Figures 7A, B; pink waveforms). Similarly, in hCICs, the minimum RMSE_AP_ decreased from 87% to 54% when expressing I_K1_, and the minimum RMSE_CaT_ decreased from 79% to 38% when expressing I_K1_ (Figures 7C, D; pink waveforms). The corresponding raw ion channel parameter values to achieve minimum RMSE and their relevant experimental metrics are shown in Supplementary Table S1.

**FIGURE 8:**
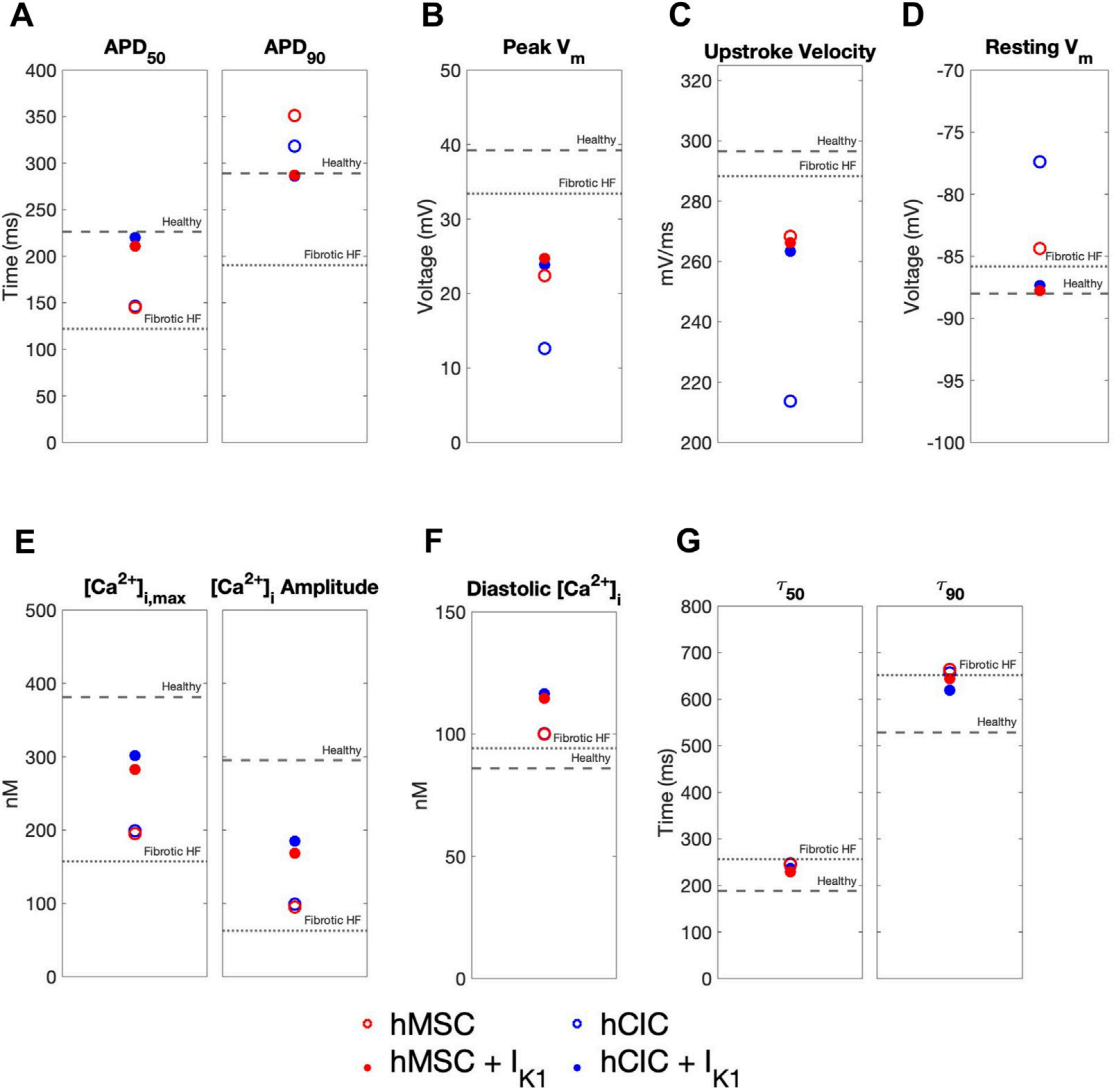
Effects of custom engineered human mesenchymal stem cell and c-kit^+^ cardiac interstitial cell heterocellular coupling on fibrotic heart failure cardiomyocyte electrophysiology and calcium handling. Action potential and calcium transient metrics are shown for coupling fibrotic heart failure (HF) cardiomyocytes to human mesenchymal stem cells (hMSCs) and human c-kit^+^ cardiac interstitial cells (hCICs) (red and blue circles, respectively) with and without cardiomyocyte inward rectifier channel (I_K1_) expression (filled and unfilled, respectively) that achieved minimum total root mean square error of action potential and calcium transient waveforms (see pink waveforms in Figure 7). Action potential metrics include: **(A)** Action potential duration (APD) at 50% repolarization (APD_50_) and 90% repolarization (APD_90_); **(B)** peak membrane voltage (V_m_); **(C)** upstroke velocity; and **(D)** resting V_m_. Calcium transient metrics include: **(E)** peak intracellular calcium and calcium transient amplitude; **(F)** diastolic calcium; and **(G)** calcium relaxation time constant at 50% decay (τ_50_) and 90% decay (τ_90_). Values for healthy and untreated fibrotic HF cardiomyocytes are shown as dashed and dotted lines, respectively.

We further explored the effects of these custom engineered hMSCs and hCICs achieving minimum total RMSE (see Figure 7 pink waveforms) on single-cell metrics associated with arrhythmogenesis—namely APD, resting membrane potential, and upstroke velocity. As shown in Figure 8, hMSCs and hCICs expressing I_K1_ had APD_90_, APD_50_, and therefore APD triangulation closer to healthy conditions (Figure 8A). In addition, resting membrane potential better resembled healthy conditions (Figure 8C). Upstroke velocity for hCICs containing I_K1_ was increased compared to hCICs without I_K1_; upstroke velocity of hMSCs with *versus* without I_K1_ was nearly unchanged (Figure 8B).

## 4 Discussion

In this study, we leverage our flexible computational pipeline to design “enhanced'' customizable engineered cells optimized to restore healthy action potential and calcium transient waveforms from failing human cardiomyocytes *via* heterocellular coupling. First, we demonstrated that candidate custom cells composed of standard non-excitable stem or interstitial cell channels were insufficient to return a failing phenotype toward healthy conditions. Next, we identified custom cells that also express cardiomyocyte ion channels and satisfied the acceptance criteria to correct fibrotic HF cardiomyocytes, though they were ineffective for correcting non-fibrotic HF hCMs. We then showed that specific cardiomyocyte ion channels are critical for this correction, whereby incorporating the inward rectifier channel minimized RMSE as well as action potential single-cell metrics associated with arrhythmogenesis. Mechanistically, we found accepted custom cells increase cardiomyocyte L-type calcium activity and thereby enhance calcium-induced calcium release. Finally, to investigate possible methods for practical implementation of the approach, we demonstrated that heterocellular coupling of simulated hMSCs and hCICs genetically engineered to express the cardiomyocyte inward rectifier K^+^ channel led to a substantial decrease in RMSE_AP_ and RMSE_CaT_ with healthy restoration of action potential single-cell metrics associated with arrhythmogenesis, indicating improved correction of fibrotic HF cardiomyocytes compared to unmodified hMSCs and hCICs. Altogether, our work demonstrates that computational modeling facilitates exploration of customizable engineered therapeutic cells and theoretically provides evidence for expressing cardiomyocyte I_K1_ on hMSCs and hCICs for improved cellular therapeutics, providing an avenue for further experimental exploration.

### 4.1 Comparison to previous empirical findings

The concept of delivering somatic cells genetically engineered to exert a specific electrical influence on host cardiomyocytes has been previously hypothesized and experimentally investigated (Yankelson et al., 2008; Kirkton and Bursac, 2012; McSpadden et al., 2012; Nussinovitch et al., 2014; Kostecki et al., 2021). Several proof-of-concept studies have demonstrated the capability of engineered cells to alter cardiomyocyte AP, CaT, and arrhythmogenesis. For example, Yankelson et al. showed that mouse fibroblasts genetically engineered to express inward or delayed rectifier potassium channels hyperpolarized resting membrane potential, reduced action potential amplitude, and prolonged the cardiac effective refractory period when delivered to pig myocardium (Yankelson et al., 2008). The Bursac group later showed that coupling genetically engineered human embryonic kidney cells with variable inward rectifier and gap junction channel expression led to distinct effects on cardiac AP shape and initiation (McSpadden et al., 2012). These findings motivated more recent work by Kirton and Bursac, where delivering genetically engineered human embryonic kidney cells expressing inward rectifier, sodium, and gap junction channels in an *in vitro* model of fibrotic neonatal rat myocardium led to improved twitch force amplitude (Kirkton and Bursac, 2012).

These experimental findings complement our computer simulations. For example, interestingly our genetic optimization algorithm also identified cardiomyocyte inward rectifier channel as critical to improving AP and CaT waveforms in failing hCMs. The expression of these channels in custom cells also led to hyperpolarized resting membrane potential, reduced action potential amplitude, and higher calcium transient amplitude (a surrogate of twitch force).

### 4.2 Translational relevance and significance

Our findings provide theoretical insight into approaches to improve translational cell-based cardiac therapies. First, we demonstrate that custom cells that express cardiomyocyte ion channels are capable of correcting failing cardiomyocyte behavior, and that expression of cardiomyocyte inward rectifier and gap junction connexin-43 activity may be sufficient to accomplish this. These findings complement ongoing investigations; for example, the Bursac group has recently created a pipeline to generate and customize cell-based therapies (Nguyen et al., 2018), and has demonstrated that primary human fibroblasts expressing sodium channels, an inward-rectifying potassium channel, and connexin-43 can rescue conduction slowing in an *in vitro* model of cardiac interstitial fibrosis (Nguyen et al., 2016). However, in contrast to these studies, our custom cells decreased upstroke velocity (as our acceptance criteria were RMSE of AP and CaT, leading to phase 0/upstroke velocity not being prioritized in the genetic algorithm). This may be mitigated if custom cells also incorporated sodium channels (like the aforementioned experimental studies), a potential area of future investigation.

Second, our findings whereby custom engineered hMSCs and hCICs expressing inward rectifier channels reduced the RMSE in failing cardiomyocytes provide an avenue for improving current cell-based therapeutics. Predominant hMSC and hCIC cardiotherapeutic mechanisms of action involve paracrine signaling and heterocellular coupling (Menasche, 2018). As shown in our recent simulations, heterocellular coupling alone in standard hMSCs and hCICs was insufficient to correct a failing hCM phenotype (Phillips et al., 2021), motivating the current effort to design enhanced cell-based therapeutics. Our simulations suggest that incorporation of an inward rectifier channel improves a failing cardiac phenotype, and may minimize arrhythmogenic risk based on our single-cell metrics; these findings motivate future efforts to experimentally test custom hMSCs, hCICs, or CardioChimeras expressing an inward rectifier channel to optimize heterocellular coupling effects of delivered cells. Notably, such heterocellular coupling should be considered in the context of established paracrine effects of these therapeutic cells (Mayourian et al., 2017; Mayourian et al., 2018b).

Third, our computational study provides a framework for optimizing other therapeutic cell types. For example, *in vivo* delivery of human induced pluripotent stem cell-derived cardiomyocytes to the failing heart remains an active area of investigation (Dhahri et al., 2022). Limitations of this modality include an immature cardiac phenotype and poor electrical integration with host tissue (Dhahri et al., 2022). Our computational framework can be readily modified to optimize ion channel expression to improve the therapeutic potential of such stem cell-derived hCMs.

### 4.3 Insights developed from electrophysiological simulations

In addition to providing avenues of future translational work, our simulations provide insight into healthy and failing cardiomyocyte electrophysiology. First, our simulations suggest L-type calcium current was more robust in fibrotic HF cells treated with accepted custom cells; this could be explained by achieving a favorable resting membrane potential to maximize channel kinetics, or by achieving a more favorable peak cardiomyocyte membrane voltage that would correspond to larger magnitude currents based on the L-type calcium channel current-voltage relationship. By increasing L-type calcium channel activity, this would presumably increase calcium-induced calcium release.

Interestingly, our candidate custom cells expressing all non-excitable and cardiomyocyte ion channels led to a larger RMSE than expressing only cardiomyocyte inward rectifier and L-type calcium channel. In the latter experiment, AP phase 1-3 outward currents are set to zero in custom cells, leading to no competing activity against inward L-type calcium channel activity; by doing so, we reveal the minimal role of L-type calcium channel needed in our accepted custom cells. Interestingly, to our surprise, nearly passive cells with minimal L-type calcium channel activity are also accepted, and passive cells alone are nearly accepted, albeit with adverse prolonged APD triangulation and APD_90_ at risk for arrhythmogenesis.

### 4.4 Optimization methodology and practical considerations

In this study, the wide range of candidate ion channels presented challenges for the unique and robust identification of optimal channels for expression in custom cells. Herein, we demonstrate the effectiveness of a genetic algorithm-based method to design custom cells that maximize HF correction. Inspired by Darwinian evolution and natural selection, genetic algorithms are an evolutionary metaheuristic method executed in a stochastic combinatorial manner (Akwaboah et al., 2021). One advantage of this method (compared to gradient-based optimization methods) is that it is less susceptible to getting stuck at local minima, often converging at the globally optimal solution even with high-dimensional parameter spaces (Gulsen et al., 1995). Similar to our approach, genetic algorithms have previously been applied to cardiac electrophysiology models using simple perturbations of maximum channel conductance to generate a desired AP morphology (Syed et al., 2005; Bot et al., 2012; Tomek et al., 2019; Smirnov et al., 2020).

To ensure biologically relevant solutions, we imposed physiologic bounds on the searchable parameter space, guided by following previous work (specifically for gap junction conductance) (Valiunas et al., 2004; Phillips et al., 2021) or by scaling established ion channel maximal conductances for non-excitable cells (Phillips et al., 2021) or cardiomyocytes (O'Hara et al., 2011; Mora et al., 2018). In addition, accepted solutions were restricted to no more than 5 coupled cells per cardiomyocyte, which would be challenging to exceed in clinical practice (although theoretically it is within the modeling capabilities).

### 4.5 Limitations and future directions

This study has some recognized limitations. First, our candidate channels were limited to previously modeled non-excitable and cardiomyocyte channels. To our knowledge, we used the only published models for hMSCs (Mayourian et al., 2016) and hCICs (Phillips et al., 2021). Other cardiomyocyte and fibroblast models are available; however, to our knowledge only the combination of the O’Hara (O'Hara et al., 2011) and MacCannell (MacCannell et al., 2007) models have been used to develop a human fibrotic HF cardiomyocyte model, prompting us to use these models. We note that hMSC, hCIC, and CF leakage currents were excluded in our genetic algorithm, as they are often mathematical constructs to match electrophysiological measurements rather than biologically relevant channels, and by study design we aimed to express physiologic channels.

Second, by experimental design, in this study we solely focused on heterocellular coupling effects of custom cells, including engineered hCICs and hMSCs. However, it is well established that hMSC and hCIC cardiotherapeutic mechanisms of action also involve paracrine signaling (Menasche, 2018). For example, previous work showed that hMSC paracrine signaling alters excitation-contraction coupling of cardiomyocytes, causing increased expression of key calcium cycling genes—including sarcoendoplasmic reticulum Ca^2+^-ATPase and L-type calcium channels—and thereby increasing cardiomyocyte calcium transient amplitude and contractility (DeSantiago et al., 2012; Mayourian et al., 2017; Mayourian et al., 2018a; Mayourian et al., 2018b). Although our recent work has modeled paracrine signaling effects in these known cell types (Phillips et al., 2021), modeling the paracrine effects of custom cells was beyond the scope of this study and would first need to be determined empirically.

Third, we only provide single-cell metrics of arrhythmogenicity, as tissue-level analysis was beyond the scope of the current study. Herein, we show that custom hMSC and hCIC cells expressing cardiomyocyte inward rectifier channel had APD_90_, APD_50_, and therefore APD triangulation closer to healthy conditions. In addition, resting membrane potential better resembled healthy conditions, all favorable single-cell indicators of arrhythmias. Nevertheless, all coupled cell models have decreased upstroke velocity, which may indicate a pro-arrhythmic potential of custom cells. Arrhythmogenic risk of hMSCs has been addressed previously by our group using tissue-level analysis (Mayourian et al., 2016; Mayourian et al., 2018a) and a similar approach could be used with the accepted custom cells and/or engineered hMSCs and hCICs. Addressing this limitation may be clinically relevant, as the recent CONCERT-HF trial showed a higher incidence of ventricular arrhythmias in all cell therapy groups, with the highest rate of occurrence reported with isolated hCIC treatment (albeit these findings were not statistically significant) (Bolli et al., 2021). Notably, our simulations suggest hCICs expressing the cardiomyocyte inward rectifier channel led to increased upstroke velocity compared to control hCICs.

Fourth, we note that the stringent acceptance criteria could lead to false negatives; for example, it is feasible that the restoration of AP or CaT alone may be sufficient to provide meaningful physiologic benefit despite not meeting our predefined acceptance criteria for both AP and CaT. However, we chose to err on the side of being conservative in these exploratory studies. In addition, given the computational expense of implementing a genetic algorithm across many parameters, we imposed bounds on the searchable space for certain parameters (e.g., gap junctional conductance) based on previous findings (Valiunas et al., 2004; Phillips et al., 2021). However, the nature of this exploratory simulation work allows for supraphysiologic conditions, which could also lead to acceptable solutions. For example, accepted custom cell heterocellular gap junctional conductances were 5–8 nS; while this is higher than reported hMSC-cardiomyocyte coupling conductances (Valiunas et al., 2004), even larger conductances that are representative of cardiomyocyte-cardiomyocyte junctional conductances (Jongsma and Wilders, 2000; Dhillon et al., 2013) may also lead to acceptable solutions. Such parameters beyond our searchable space could be more favorable in terms of resulting tissue-level metrics such as conduction velocity, warranting further investigation.

Finally, we acknowledge that although mathematical models offer the capability to explicitly control variables that can be challenging to manipulate experimentally, they are also subject to the limitations of their underlying assumptions, and ultimately the most valuable insights will come from a synergistic combination of computational modeling and experimental validation. For example, herein we assume all cardiomyocytes will need to be sufficiently restored, requiring an abundant number of therapeutic cells to be delivered (2-5 custom cells per cardiomyocyte). However, recent gene therapy studies have shown that correcting only a small percentage of cardiomyocytes can improve muscle function (Long et al., 2016; Nelson et al., 2016; Dave et al., 2022). Further *in vitro* and *in vivo* validation studies are therefore warranted to explore the cell coupling efficiency required to achieve a therapeutically beneficial restoration effect.

To support the translation of our simulated work to the bench, we provide estimations of raw ion channel parameter values required to achieve restoration. There already exists a precedent of reprogramming non-excitable cells (Zhou et al., 1998; Chen et al., 2007), hMSCs (Bruzauskaite et al., 2016), and hCICs (Vegh et al., 2020) to express non-native ion channels that survive well, thus providing promise for a similar methodology. However, we acknowledge the practical challenge of titrating the expression of channels of interest to match the accepted range of solutions listed herein; for convenience, we provide practical reference data points for wet lab colleagues to validate our computational findings and potentially enhance cell-based cardiotherapies.

## Data Availability

The raw data supporting the conclusions of this article will be made available by the authors, without undue reservation.

## Abbreviations

AP, Action potential; APD_x_, Action potential duration at x% repolarization; CaT, calcium transient; C_CM_, Cardiomyocyte membrane capacitance; C_custom_, Custom cell membrane capacitance; [Ca^2+^]_i_, Intracellular calcium; [Ca^2+^]_i,max_, Maximum intracellular calcium; CF, Cardiac fibroblast; G_x_, Conductance of channel x; G_gap_, Gap junctional conductance between cells; hCIC, Human c-kit^+^ cardiac interstitial cell; hCM, Human cardiomyocyte; HF, Heart failure; hMSC, Human mesenchymal stem cell; I_CaL_, Cardiac L-type Ca^2+^ current; I_ion, CM_, Total ionic flow through cardiomyocyte; I_ion, custom_, Total ionic flow through custom cell; I_K1_, Inward rectifier K^+^ current; IQR, interquartile range; n_custom_, Number of coupled custom cells; RMSE, Root mean square error; τ_x_, Calcium relaxation time constant at x% decay; V_CM_, Cardiomyocyte membrane potential; V_custom_, Custom cell membrane potential.
