# Supplementary material for: Computational design of custom therapeutic cells to correct failing human cardiomyocytes
Source: Front Syst Biol. 2023 Jan 18;3:1102467. doi: 10.3389/fsysb.2023.1102467 (PMC9894098; doi:10.3389/fsysb.2023.1102467)
Supplement: Supplementary file 1 [file DataSheet1.pdf]

Supplementary Material

1 Supplementary Figures

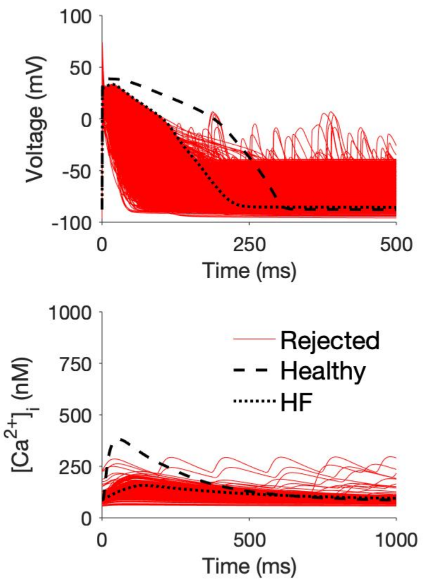

Supplementary Figure 1: Custom cells lacking two key excitable cell channels were unable to correct the phenotype of fibrotic failing cardiomyocytes. Initial populations of 2500 custom cell models were generated for heterocellular coupling with fibrotic heart failure (HF) cardiomyocytes with random perturbations of gap junction conductances, number of coupled cells, and both cardiomyocyte and non-excitable cell ion channel maximal conductivities, only excluding cardiomyocyte L-type calcium and inward rectifier channel currents. No custom cells achieved root mean square error less than 50% for both action potential and calcium transient waveforms, and all were therefore rejected (red). Waveforms for healthy and untreated fibrotic HF cardiomyocytes are indicated by dashed and dotted lines, respectively.

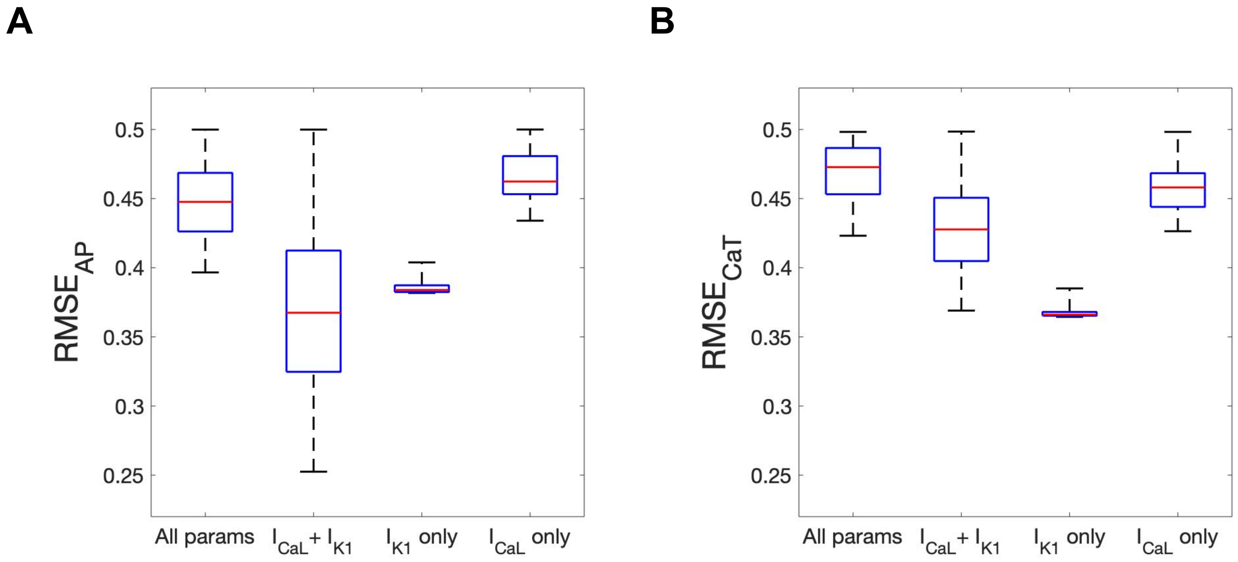

Supplementary Figure 2: Root mean square error for accepted custom cells. The root mean square error (RMSE) was calculated for action potential ($RMSE_{AP}$) and calcium transient ($RMSE_{CaT}$) waveforms for the following experiments: 1) custom cells expressing non-excitable cell ion channels and cardiomyocyte channels (All params); 2) custom cells expressing only cardiomyocyte L-type calcium and inward rectifier channels (I_{CaL} + I_{K1}); 3) custom cells expressing only cardiomyocyte inward rectifier channels (I_{K1}); and 4) custom cells expressing only cardiomyocyte L-type calcium channels (I_{CaL}).

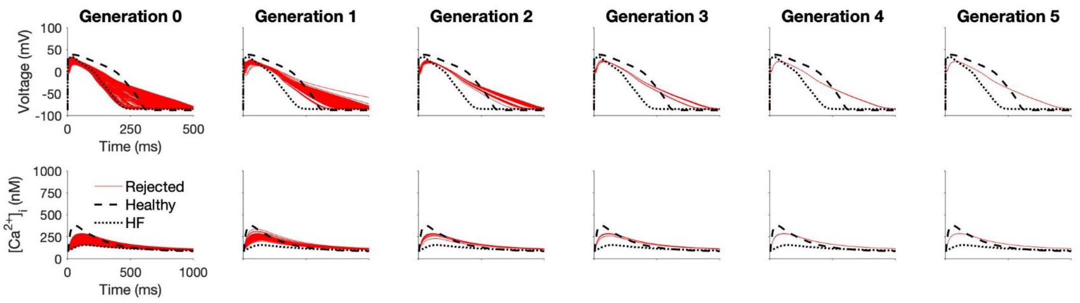

Supplementary Figure 3: **Genetic algorithm-based approach demonstrates passive cells are unable to restore failing cardiomyocyte phenotype.** Initial populations of 2500 passive cell models were generated for heterocellular coupling with fibrotic heart failure (HF) cardiomyocytes with pseudo-random perturbations of gap junction conductances and number of coupled cells. Passive cell populations underwent 5 generations of genetic algorithm evolution. No passive cells were accepted with root mean square error <50% for both action potential and calcium transient waveforms; all models outside this range were rejected (red). Waveforms for healthy and untreated HF cardiomyocytes are indicated by dashed and dotted lines, respectively.

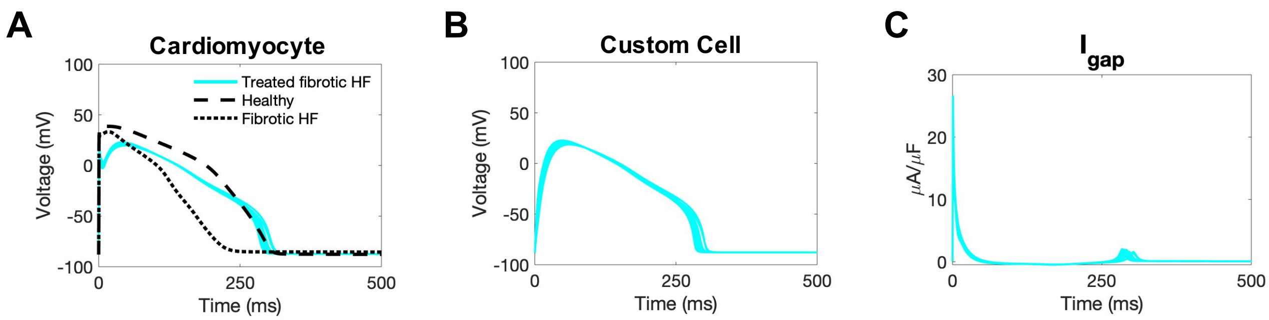

Supplementary Figure 4: Sinking effects of accepted custom cells on fibrotic heart failure cardiomyocytes. Fibrotic heart failure (HF) cardiomyocytes when coupled to 25 randomly selected accepted custom cell solutions (cyan) that express cardiomyocyte inward rectifier channel. (**A**) Treated fibrotic HF cardiomyocyte (cyan) action potential, compared to healthy (dashed) and untreated HF (dotted) cardiomyocytes. (**B**) 25 randomly selected accepted custom cell membrane voltages. (**C**) Gap junction current density (I_{gap}) between the treated HF cardiomyocyte and the coupled accepted custom cell; positive I_{gap} corresponds to positive flow out of the myocyte and into the coupled accepted custom cell.

2 Supplementary Tables

Supplementary Table 1: Parameters for Accepted Custom Cell Models

Model	n_{HC}	G_{junct} (nS)*	G_{CaL} (cm/s)	Peak I_{CaL} (pA/pF)**	G_{K1} (nS)	I_{K1} at -120 mV (pA/pF)***
I_{CaL} + I_{K1}	4 [3,4]	7.3 [5.5,8.7]	3 [2,7] x 10^{-6}	-1.1 [-0.8,-2.7]	0.23 [0.14,0.40]	-61 [-35,-105]
I_{K1}-only	5 [5,5]	5.7 [5.3,6.1]	—	—	0.24 [0.22,0.25]	-62 [-57,-66]
I_{CaL}-only	4 [4,4]	7.8 [6.5,9.2]	3 [2,4] x 10^{-6}	-1.1 [-0.8,-1.5]	—	—
hMSC	2	9.6	—	—	—	—
hMSC+ I_{K1}	3	4.0	—	—	0.44	-113
hCIC	2	15.0	—	—	—	—
hCIC + I_{K1}	5	3.3	—	—	0.33	-87

Data presented as median [25th percentile, 75th percentile] when applicable.

*In comparison to cardiomyocyte-cardiomyocyte gap junctional conductance of approximately 1000 nS based on left ventricular gap junction resistivity[1] and its correlation to simulated cardiomyocyte gap junctional conductance.[2]

**Using voltage-clamp protocol from Figure 1 of O'Hara et al.[3]

***Using voltage-clamp protocol from Figure 2 of O'Hara et al.[3]

3 References

1 Dhillon, P.S., Gray, R., Kojodjojo, P., Jabr, R., Chowdhury, R., Fry, C.H., et al. (2013). Relationship between gap-junctional conductance and conduction velocity in mammalian myocardium. *Circ Arrhythm Electrophysiol* 6**,** 1208-1214.

2 Jongsma, H.J., and Wilders, R. (2000). Gap junctions in cardiovascular disease. Circ Res 86, 1193-1197.

3 O'hara, T., Virag, L., Varro, A., and Rudy, Y. (2011). Simulation of the undiseased human cardiac ventricular action potential: model formulation and experimental validation. PLoS Comput Biol 7, e1002061.
